# A deep learning-based computer-aided diagnosis system for detecting atypical endometrial hyperplasia and endometrial cancer through hysteroscopy

**DOI:** 10.1016/j.isci.2025.113045

**Published:** 2025-07-03

**Authors:** Wenwen Wang, Yuyang Cai, Zhe Guo, Aihua Zhao, Wenqing Ma, Wuliang Wang, Shixuan Wang, Xin Zhu, Xin Du, Wenfeng Shen

**Affiliations:** 1 Department of Obstetrics and Gynecology, Tongji Hospital, Tongji Medical College, Huazhong University of Science and Technology, Wuhan, China; 2 School of Computer and Information Engineering, Shanghai Polytechnic University, Shanghai, China; 3 Graduate School of Computer Science and Engineering, The University of Aizu, Aizu-Wakamatsu, Japan; 4 Department of Obstetrics and Gynecology, The Second Affiliated Hospital of Zhengzhou University, Zhengzhou, Henan, China; 5 Department of Laboratory Medicine, The Second Xiangya Hospital, Central South University, Changsha, China; 6 Department of AI Technology Development, M&D Data Science Center, Institute of Integrated Research, Institute of Science Tokyo, Tokyo, Japan; 7 Department of Gynecology, Maternal and Child Health Hospital of Hubei Province, Tongji Medical College, Huazhong University of Science and Technology, Wuhan, China; 8 College of Virtual Reality Modern Industry, Jiangxi University of Finance and Economics, Nanchang, Jiangxi, China

**Keywords:** Computer-aided diagnosis method, Cancer, Machine learning

## Abstract

Timely diagnosis of endometrial cancer (EC) and atypical endometrial hyperplasia (AEH) is crucial, yet traditional hysteroscopy faces accuracy challenges. This study introduces ECCADx, a deep learning-based computer-aided diagnosis system utilizing contrastive learning for hysteroscopic identification of AEH and EC. This is the system to integrate contrastive learning for this specific differentiation. ECCADx leveraged contrastive learning during pre-training on diverse external medical images, extracting robust features. Trained on 49,646 images from 1,204 patients, it underwent rigorous multicenter validation on two independent test datasets (6,228 images from 190 patients). ECCADx consistently achieved high diagnostic accuracy, often surpassing experienced endoscopists. Notably, it attained 95.2% sensitivity and 91.3% specificity on the internal dataset, and 92.1% sensitivity with 100% specificity on the external dataset. ECCADx proves a reliable tool, comparable or superior to human experts, promising to reduce misdiagnosis and improve patient outcomes.

## Highlights

• AI system ECCADx assisting doctors in detecting AEH/EC using hysteroscopy
• ECCADx outperforms endoscopists in diagnosing endometrial lesions
• Model robustly detects lesions across hospital hysteroscopy images
• Contrastive learning and domain-specific augmentation enhance adaptability

## Graphical abstract

**Figure:**
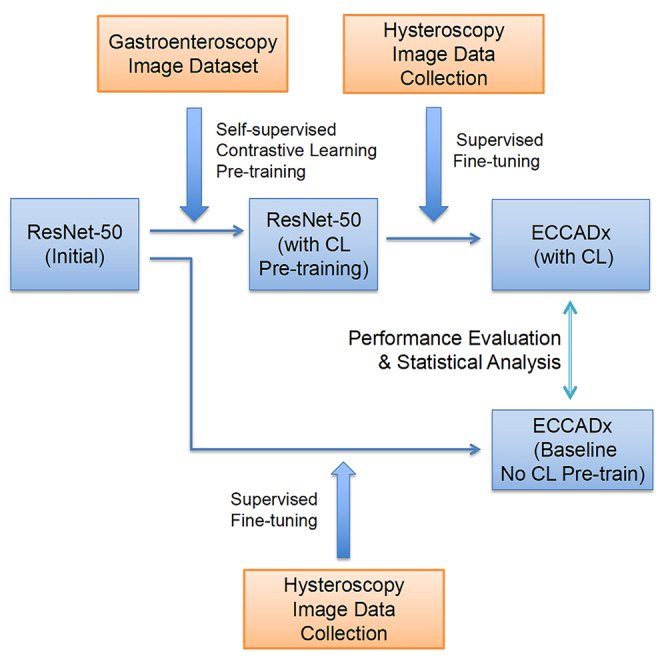

## Introduction

As a common malignant gynecological condition, the global incidence of endometrial cancer (EC) reached 417,336 cases in 2020, making it the sixth most common cancer among women.^1^ While 67% of patients are diagnosed at an early stage, with a 5-year overall survival (OS) rate of 81%, the 5-year OS drops significantly to 17% for stage IVA, and 15% for stage IVB EC.^2^ As a precursor lesion of EC, atypical endometrial hyperplasia (AEH) carries a high progression risk at 28% over 20 years^3^ In addition to its increased progression risk, AEH may coexist with occult EC in up to one-third of cases.^4^ Hence, early diagnosis of EC and AEH and intervention are crucial.

Hysteroscopic-guided curettage is increasingly recognized as an effective method for customizing treatment plans for patients with EC. Studies have shown that hysteroscopy offers superior diagnostic capabilities compared to dilation and curettage (D&C) performed alone.^5^ A comprehensive meta-analysis conducted by Gkrozou et al., which included data from over 9,000 patients, evaluated the diagnostic accuracy of hysteroscopy for conditions such as polyps, submucosal myomas, hyperplasia, and EC. The results indicated that hysteroscopy achieves a high level of accuracy in diagnosing EC, with a sensitivity of 82.6% and a specificity of 99.7%.^6^

However, misdiagnosis can lead to an underestimation of the risk associated with uterine conditions, resulting in treatment delays. A recent meta-analysis involving 1106 patients with a preoperative diagnosis of atypical endometrial hyperplasia revealed that uterine curettage and hysteroscopic-guided biopsy underestimated the presence of EC in 32.7%–45.3% of cases.^7^ Similarly, another systematic review and meta-analysis assessing the diagnostic performance of D&C and hysteroscopy in postmenopausal women with bleeding found an 11% failure rate and 31% infeasible endometrial samples, resulting in missed diagnoses in approximately 7% of cases.^8^ Given the significant risks posed by missed diagnoses, there is an urgent need for improved diagnostic tools to enhance the accuracy of EC evaluation.

In recent years, deep learning has gained widespread use in endoscopic procedures, particularly for detecting conditions, such as polyps, adenomas, and gastrointestinal cancers using colonoscopy, gastroscopy, hysteroscopy, and so on.^9^^,^^10^^,^^11^ Contrastive learning (CL), a robust deep learning approach, has demonstrated significant potential in enhancing model performance by enabling the learning of more discriminative and resilient features.^12^ This method has been successfully employed in various medical image analysis tasks, improving the model’s ability to identify subtle distinctions in medical images, which in turn boosts diagnostic accuracy and reliability. This study presents a deep learning-based computer-aided diagnosis system (ECCADx) for differentiating AEH and EC from benign lesions specifically in hysteroscopic images, addressing a significant clinical unmet need.

Several studies have demonstrated the effectiveness of CL in various medical imaging applications. Chaitanya et al. successfully applied CL to improve the segmentation of medical images, particularly in MRI and CT scans, by leveraging both global and local features to enhance model performance in scenarios with limited annotations.^13^ Sowrirajan et al. further extended the application of CL by introducing MoCo-CXR, a method that significantly improved the representation quality and transferability of models used for chest X-ray interpretation, showing notable benefits, especially when labeled data were scarce.^14^ Most recently, Liu et al. applied CL in renal pathological image classification, achieving superior performance in distinguishing complex tissue structures, which underscores the growing utility of this technique in diverse medical imaging tasks.^15^

Our study introduces an application of self-supervised CL for pre-training in hysteroscopic image analysis in this specific domain for EC and atypical hyperplasia detection. This innovative approach addresses the inherent challenge of limited large-scale annotated hysteroscopy datasets by leveraging diverse endoscopic imaging data (from colonoscopy) to learn robust, transferable visual features. By allowing the model to acquire rich low-level and mid-level representations without manual annotations, this strategy significantly enhances ECCADx’s ability to generalize and accurately identify subtle pathological changes, setting a new precedent for deep learning in hysteroscopic diagnostics.

While colonoscopy and hysteroscopy images originate from distinct anatomical sites, they share fundamental characteristics as endoscopic images, such as mucosal patterns, vascular structures, and the appearance of pathological lesions (e.g., polyps, masses). Both modalities face similar imaging challenges, including variable illumination, specular reflections, and limited fields of view. However, significant anatomical and physiological differences exist between the colon and the uterine endometrium, leading to variations in normal mucosal appearance, color, texture, and specific lesion morphologies. For instance, the villous structures in the colon differ from the endometrial glands, and the typical presentation of colorectal polyps may vary from endometrial polyps or hyperplasias. Despite these differences, the underlying visual patterns of disease progression (e.g., abnormal vascularity, irregular surface structures, and mass formation) can exhibit certain generalizable features across various endoscopic examinations.

Currently, studies on the use of hysteroscopy for endometrial lesion identification are single-center studies, which limit the broader application of the findings.^10^ To address this gap, Addressing the limitations of existing single-center studies, our research marks a breakthrough with its multicohort retrospective validation across three tertiary hospitals, offering robust evidence of ECCADx’s generalizability and clinical applicability. This rigorous external validation, using diverse patient populations and equipment, is a critical step toward real-world adoption. This system, based on deep learning and CL techniques, aims to accurately identify AEH and EC from benign lesions.^16^

In this study, we performed a multicohort retrospective analysis of 1394 cases from three tertiary hospitals to develop and validate the EC computer-aided diagnosis (ECCADx) system. This system leverages deep learning and CL techniques to accurately identify AEH and EC from benign lesions, offering a significant improvement over existing diagnostic methods. Our comprehensive multi-center external validation significantly enhances the reliability and trustworthiness of ECCADx’s performance.

## Results

### Performance of models on test datasets

**Figure 1:**
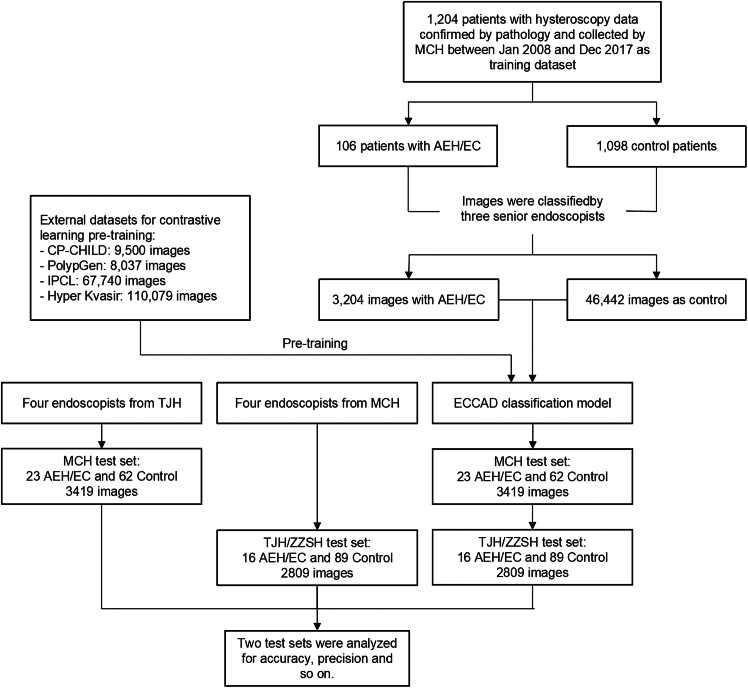
Flowchart on development and validation of ECCADx

ECCADx was trained and used as illustrated in Figure 1 to estimate the performance of the proposed model on two test datasets listed in Table 1. For the MCH test dataset, The ECCADx without CL achieved an Area Under the Curve (AUC) value of 0.969 (95% confidence interval (CI): 0.928–0.999), accuracy of 91.8% (95% CI: 85.9%–97.6%), sensitivity of 96.8% (95% CI: 91.7%–100%), specificity of 78.3% (95% CI: 60.9%–92.9%), and an F1 score of 0.945 (95% CI: 0.902–0.979). In contrast, the ECCADx with CL demonstrated slightly improved performance, with an AUC value of 0.979 (95% CI: 0.942–1.000), accuracy of 94.1% (95% CI: 89.1%–99.1%), sensitivity of 95.2% (95% CI: 89.5%–100%), specificity of 91.3% (95% CI: 78.2%–100%), and an F1 score of 0.959 (95% CI: 0.920–0.992) in Table 2. For the TJH/ZZSH test dataset, The ECCADx without CL achieved an AUC value of 0.891 (95% CI: 0.810–0.964), accuracy of 89.5% (95% CI: 83.7%–95.4%), sensitivity of 94.4% (95% CI: 89.4%–98.8%), specificity of 62.5% (95% CI: 40.0%–86.7%), and an F1 score of 0.939 (95% CI: 0.898–0.973). In contrast, the ECCADx with CL demonstrated significantly improved performance, with an AUC value of 0.975 (95% CI: 0.942–0.998), accuracy of 93.3% (95% CI: 88.6%–98.1%), sensitivity of 92.1% (95% CI: 86.4%–96.8%), specificity of 100% (95% CI: 100-100%), and an F1 score of 0.959 (95% CI: 0.925–0.988) in Table 3.

**Table 1:** Baseline characteristics of training and test datasets

	MCH training dataset		MCH test dataset		TJH/ZZSH test dataset	
	AEH/EC^a^	Control	AEH/EC	Control	AEH/EC	Control
Cases	106	1098	23	62	16	89
Images	3204	46,442	698	2721	760	2049

a AEH/EC: endometrial atypia hyperplasia and endometrial cancer.

**Table 2:** Per patient diagnostic performance of endoscopists versus ECCADx in the MCH test dataset

	Gynecological endoscopist			ECCADx (without CL^a^)	ECCADx (with CL)
	Junior Endoscopists	Medium Endoscopists	Senior Endoscopists		
AUC (95% CI)	0.872 (0.794–0.938)	0.934 (0.877–0.975)	0.952 (0.902–0.986)	0.969 (0.928–0.999)	0.979 (0.942–1.000)
Accuracy (95% CI)	83.5% (75.7–91.4%)	87.9% (81.1–94.8%)	90.9% (84.9–96.9%)	91.8% (85.9–97.6%)	94.1% (89.1–99.1%)
Sensitivity (95% CI)	73.9% (56.9–88.1%)	72.8% (55.0–88.6%)	87.0% (77.5–95.4%)	96.8% (91.7–100%)	95.2% (89.5–100%)
Specificity (95% CI)	87.1% (78.5–94.5%)	93.5% (86.9–98.8%)	92.3% (85.3–98.0%)	78.3% (60.9–92.9%)	91.3% (78.2–100%)
PPV (95% CI)	68.2% (49.3–86.3%)	80.9% (62.2–96.0%)	80.8% (63.6–96.2%)	92.3% (84.9–98.4%)	96.7% (91.8–100%)
NPV (95% CI)	90.5% (83.0–96.6%)	90.5% (83.5–96.6%)	95.3% (91.6–98.5%)	90.0% (75.0–100%)	87.5% (72.7–100%)
F1 (95% CI)	0.701 (0.526–0.829)	0.761 (0.607–0.884)	0.833 (0.702–0.931)	0.945 (0.902–0.979)	0.959 (0.920–0.992)
Kappa (95% CI)	0.588 (0.397–0.767)	0.681 (0.488–0.844)	0.770 (0.606–0.907)	0.782 (0.601–0.916)	0.853 (0.712–0.969)
Brier (95% CI)	0.143 (0.112–0.177)	0.103 (0.076–0.133)	0.081 (0.051–0.115)	0.060 (0.033–0.093)	0.040 (0.014–0.075)

a CL, Contrastive Learning.

**Table 3:** Per patient diagnostic performance of endoscopists versus ECCADx in the TJH/ZZSH test datasets

	Gynecological endoscopist			ECCADx (without CL)	ECCADx (with CL)
	Junior Endoscopists	Medium Endoscopists	Senior Endoscopists		
AUC (95% CI)	0.773 (0.659–0.868)	0.742 (0.609–0.855)	0.862 (0.783–0.929)	0.891 (0.810–0.964)	0.975 (0.942–0.998)
Accuracy (95% CI)	76.7% (68.6–84.7%)	77.4% (69.5–85.3%)	80.2% (72.7–87.8%)	89.5% (83.7–95.4%)	93.3% (88.6–98.1%)
Sensitivity (95% CI)	65.6% (44.8–84.4%)	57.8% (35.3–79.9%)	71.9% (49.2–92.0%)	94.4% (89.4–98.8%)	92.1% (86.4–96.8%)
Specificity (95% CI)	78.7% (69.9–86.6%)	80.9% (72.7–88.6%)	81.7% (73.6–88.9%)	62.5% (40.0–86.7%)	100% (100.0–100%)
PPV (95% CI)	35.8% (17.8–55.1%)	35.8% (18.3–55.0%)	43.5% (25.1–63.2%)	93.3% (87.9–97.8%)	100% (100.0–100%)
NPV (95% CI)	93.2% (87.6–97.7%)	91.5% (85.1–96.9%)	94.3% (88.8–98.7%)	66.7% (40.0–88.2%)	69.6% (50.0–87.5%)
F1 (95% CI)	0.448 (0.249–0.622)	0.438 (0.248–0.604)	0.530 (0.329–0.698)	0.939 (0.898–0.973)	0.959 (0.925–0.988)
Kappa (95% CI)	0.319 (0.114–0.508)	0.308 (0.140–0.511)	0.419 (0.205–0.608)	0.584 (0.330–0.780)	0.781 (0.607–0.926)
Brier (95% CI)	0.184 (0.151–0.219)	0.166 (0.137–0.197)	0.157 (0.121–0.200)	0.103 (0.068–0.141)	0.072 (0.044–0.109)

Other evaluation metrics such as positive predictive value (PPV), negative predictive value (NPV), kappa coefficient, and Brier were listed in Tables 2 and 3. Figure S1 illustrates the receiver operating characteristic (ROC) and decision curve analysis (DCA) curves of ECCADx and endoscopists in identifying AEH/EC.

### Performance of deep learning versus endoscopists

For the MCH test dataset, when comparing the average performance of junior, medium, and senior endoscopists (four per group) to the ECCADx model, the deep learning system consistently outperformed human experts. With CL, ECCADx achieved higher AUC (0.979 vs. 0.952), accuracy (94.1% vs. 87.0%), sensitivity (95.2% vs. 92.3%), and F1 score (0.959 vs. 0.770) compared to the average results of senior endoscopists. These findings highlight the superior diagnostic accuracy and consistency of ECCADx, particularly when using CL.

For the TJH/ZZSH test dataset, In comparison to the average performance of TJH experts (across four individuals per group), the ECCADx model, particularly with CL, consistently demonstrated superior diagnostic accuracy. With the integration of CL, ECCADx achieved an AUC of 0.975, significantly higher than the experts’ average of 0.862. Similarly, ECCADx surpassed the experts in terms of accuracy (93.3% vs. 80.2%) and sensitivity (92.1% vs. 71.9%). Moreover, the model’s F1 score (0.959) far exceeded that of the experts (0.530), underscoring ECCADx’s enhanced consistency and precision in diagnostic performance.

**Figure 2:**
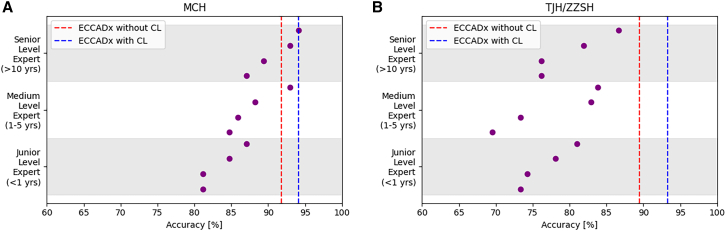
Comparison of accuracy between ECCADx models and experts of varying experience levels Validation of accuracy based on the MCH test dataset (A) and the TJH/ZZSH test datasets (B), respectively.

Other evaluation metrics such as sensitivity, specificity, negative predictive value, and Kappa coefficient for ECCADx and endoscopists were also listed in Tables 2 and 3. Regarding the comparison of accuracy between ECCADx and experts at different experience levels, as shown in Figure 2. For detailed performance of each expert, please refer to Tables S1–S6.

### Feature visualization via t-SNE

**Figure 3:**
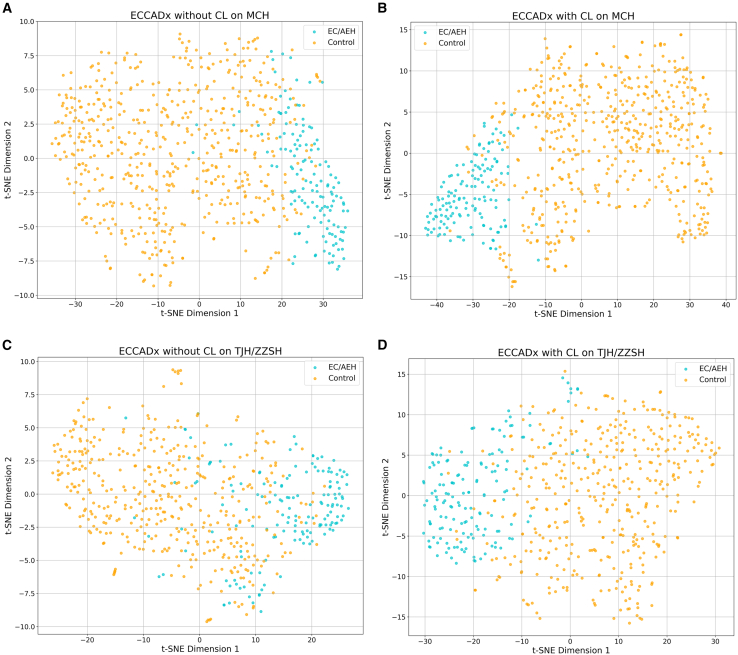
t-SNE visualization of learned feature representations from different models on internal and external test datasets (A) MCH dataset without contrastive learning. (B) MCH dataset with contrastive learning. (C) TJH/ZZSH datasets without contrastive learning. (D) TJH/ZZSH datasets with contrastive learning.

To comprehensively evaluate the feature learning capabilities and generalization abilities of our proposed ECCADx system, and to understand the impact of the CL module, we performed t-SNE (t-distributed stochastic neighbor embedding) visualization on the high-dimensional features extracted by different model configurations. t-SNE is a non-linear dimensionality reduction technique that maps high-dimensional data points onto a 2D plane, preserving local neighborhood structures to visually represent the clustering patterns of different categories. For all visualizations presented in Figure 3, orange points represent control samples, while blue points represent EC/AEH (endometrial cancer/AEH) samples.

Figure 3A (ECCADx without CL on MCH) and Figure 3C (ECCADx without CL on TJH/ZZSH) both show significant intermixing between the orange (control) and blue (EC/AEH) samples, indicating poor feature separation. The clusters are diffuse and heavily overlap, particularly in the central regions, suggesting that without CL, the model struggles to learn discriminative features for both internal and external datasets

In contrast, Figure 3B (ECCADx with CL on MCH) and Figure 3D (ECCADx with CL on TJH/ZZSH) demonstrate a remarkable improvement. Both figures show the orange and blue clusters to be far more distinct, compact, and well-separated. Although Figure 3D (external dataset) might have slightly more subtle boundaries than Figure 3B (internal dataset), the overall reduction in inter-class overlap is evident. This visually confirms that the integration of CL significantly enhances the model’s ability to extract highly discriminative and robust features, improving its performance and generalization across different clinical data sources.

Collectively, these t-SNE visualizations provide strong qualitative evidence that the CL component in ECCADx is crucial for learning highly discriminative features, which translates to superior classification performance and robust generalization across diverse clinical datasets.

## Discussion

The results confirm that ECCADx, particularly with the integration of CL, outperforms endoscopists of varying experience levels in identifying AEH/EC patients across test datasets from different medical centers. ECCADx demonstrates superior sensitivity and specificity, particularly in distinguishing AEH/EC from other non-cancerous lesions such as polyps, submucosal uterine leiomyomas, endometrial hyperplasia without atypia, and normal uterine cavities. The incorporation of CL significantly enhances the model’s feature extraction capabilities, enabling it to recognize subtle yet critical differences in complex medical images. This improvement translates to higher diagnostic accuracy, robustness, and consistency across diverse datasets. By integrating ECCADx with hysteroscopy systems, the diagnostic process can be expedited, ensuring a balanced performance among endoscopists regardless of their experience level. Additionally, ECCADx serves as a reliable assistant, reducing the likelihood of misdiagnosis and unnecessary biopsies by mitigating perceptual biases and visual fatigue commonly faced by endoscopists.

The effectiveness of CL in our study comes from its ability to enhance feature extraction.^12^ It helps the model differentiate samples better, especially with limited and imbalanced labeled data.^13^^,^^14^^,^^15^ We used colonoscopy datasets, which share similarities with hysteroscopy, to pre-train the ECCADx system. This helped address the limitation that ResNet-50 pretrained weights mainly come from non-endoscopic images.^17^ As a result, the model captured subtle differences between AEH/EC and benign lesions more effectively. This led to significant improvements in diagnostic accuracy and robustness, even on external datasets from different medical centers.

**Figure 4:**
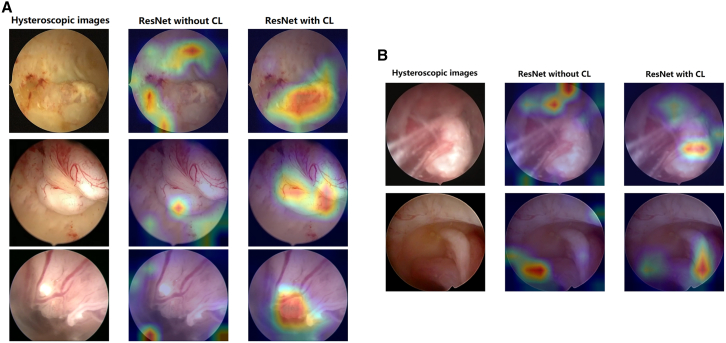
Grad-CAM visualization of ECCADx predictions for AEH and EC with and without contrastive learning (CL) (A) Correctly identified samples. This panel shows three correctly identified AEH/EC samples. (B) Misclassified false positive samples from the MCH dataset. This panel shows two false positive samples from the MCH dataset.

The Grad-CAM algorithm was employed to identify critical regions used by ECCADx for predicting AEH/EC, as demonstrated in Figure 4.^18^ The heatmaps presented in this figure highlight areas of the hysteroscopic images that may contain significant morphological and vascular features. These features, such as gross distortion of the endometrial cavity, focal necrosis, friable consistency, and atypical vessels, are linked to various pathological patterns of AEH and EC.^19^ The comparison between the models with and without CL shows that the model enhanced with CL more effectively focuses on these crucial regions. This suggests that CL significantly improves the ECCADx system’s ability to recognize and differentiate AEH/EC, by enhancing its sensitivity to these key pathological features. More images can be found in Figure S2.

In the MCH test dataset, two false negative cases of ECCADx were observed, specifically one case with polyp cystic degeneration and another with papillary proliferation. Further investigation into these hysteroscopically confirmed false negative cases revealed specific visual characteristics that notably contributed to misclassification by ECCADx, as illustrated in Figure 4B. For instance, cases exhibiting polypoid cystic degeneration presented with smooth, translucent surfaces under hysteroscopy, remarkably mimicking benign endometrial polyps. The absence of overtly irregular vasculature or distinct surface necrosis consequently reduced the model’s confidence in malignancy detection for these instances. Similarly, foci of papillary proliferation showed fine, delicate fronds with uniform coloration, appearing highly similar to surrounding healthy endometrium. Their subtle architectural disruption and lack of pronounced hyper-vascular patterns ultimately failed to trigger the model’s malignancy threshold. In contrast, in the TJH/ZZSH datasets, all cases of AEH and EC were accurately identified by the ECCADx.

The development of ECCADx relied on a dataset collected over nine years from a single hospital. Factors such as limited sample size, variations in population demographics, differences in imaging devices, and inconsistent image quality could affect the model’s stability when applied to external datasets. Geographically and temporally distinct datasets from two additional hospitals were used as external test data to evaluate the model’s classification performance. The results demonstrated ECCADx’s ability to accurately process images from various devices with differing quality and subject distributions. This variation is significantly influenced by inherent image heterogeneity across different hospitals, stemming from diverse hysteroscopy machine models (e.g., Olympus vs. Karl Storz) which capture varying color renditions, image sizes, and visual nuances. Notably, ECCADx achieved 100% specificity and PPV on TJH/ZZSH for benign cases, highlighting its strong capability in avoiding false positives and accurately identifying normal features despite this inter-institutional variability.

While senior endoscopists showed lower accuracy than ECCADx on the TJH/ZZSH dataset, it is recognized that human diagnostic performance in concentrated evaluation settings can be influenced by additional factors like endoscopist fatigue or workload. Unlike human experts, an AI model maintains consistent performance without susceptibility to such human factors, which may partially explain its relative robustness in this specific external test scenario. Despite these inter-dataset differences and the potential influence of human factors, ECCADx demonstrated robust accuracy across various devices and subject distributions, ultimately surpassing experienced endoscopists in overall evaluation. This underscores its potential as a stable, reliable, and consistent diagnostic aid in diverse clinical settings.

To bridge the gap between research and clinical translation, it is crucial to consider the practical aspects of integrating ECCADx into existing clinical workflows. Given that ECCADx is built upon a fine-tuned ResNet-50 architecture, it boasts highly efficient inference speed, typically processing each hysteroscopy image within milliseconds. This rapid processing capability enables ECCADx to support real-time auxiliary diagnosis during hysteroscopic examinations, providing clinicians with immediate “second opinions” or alerts for suspicious lesions. Furthermore, the deployment requirements for ECCADx are comparable to those for running a standard ResNet-50 model, allowing for deployment on conventional medical workstations equipped with modern GPUs or dedicated edge computing devices. This combination of low computational overhead and fast inference makes ECCADx a highly practical tool for enhancing diagnostic efficiency and accuracy in routine clinical practice, potentially reducing misdiagnosis rates and unnecessary biopsies.

Machine learning has been extensively utilized in gastrointestinal endoscopy for detecting and classifying various disorders.^20^^,^^21^ However, its application in hysteroscopy with computer-aided diagnosis remains relatively underexplored. Neofytou et al. developed a computer-aided diagnosis system for the early detection of EC.^22^ The CADx system was validated using 516 regions of interest (ROIs) from 52 subjects. For ROI classification, the highest performance was achieved by combining statistical features (SFs) and gray-level difference statistics (GLDS) features with an support vector machine (SVM) classifier, resulting in an accuracy rate of 81%.^21^ Recently, Zhang et al.’s team utilized the VGG-Net-16 model to classify endometrial lesions, achieving sensitivities of 84.0%, 68.0%, 78.0%, 94.0%, and 80.0% for endometrial hyperplasia without atypia, atypical hyperplasia, EC, endometrial polyps, and submucosal myoma, respectively.^10^ Takahashi et al. introduced an automated system employing deep learning for diagnosing EC from hysteroscopic images, tested on 177 patients. By integrating three neural network models and using a continuity analysis method, their system achieved a diagnostic accuracy of 90.29%.^16^ Compared to these approaches, ECCADx exhibited superior performance in identifying EC across a larger, more diverse sample size from multiple medical centers.

As the ECCADx system is based solely on fine-tuning a standard ResNet model, its deployment cost is comparable to that of a conventional ResNet implementation. This inherent efficiency suggests that the system can be effectively deployed and operate within relatively mature and commonly available hardware environments, thereby lowering the barrier for clinical adoption. Explicitly addressing these practical considerations will further underscore ECCADx’s potential as not just a research outcome, but a viable auxiliary diagnostic tool with tangible real-world applicability.

In summary, this study introduces the ECCADx system, which enhances AEH and EC diagnosis by leveraging CL and advanced data techniques. ECCADx shows strong generalization across diverse datasets, addressing data imbalance and improving sensitivity to malignant cases. The system outperforms experienced gynecological endoscopists in challenging scenarios, demonstrating its potential to improve diagnostic accuracy and consistency in clinical practice.

### Limitations of the study

(1) Imbalanced datasets: In general, gynecological endoscopists can identify AEH and EC based on their morphological and vascular patterns. However, notable variability in inter-rater agreement among endoscopists has been observed. Specifically, in the TJH/ZZSH test dataset, diagnostic accuracy ranged from 76.2% to 86.7%. This variability may be attributed to the lower proportion of AEH/EC cases in the TJH/ZZSH dataset (16/105) compared to the MCH dataset (23/85), which posed challenges in accurately identifying malignancies. Furthermore, since the proposed model was trained exclusively on data from MCH, its performance was somewhat lower when analyzing TJH/ZZSH data. This discrepancy could be due to inter-hospital differences in factors, such as imaging equipment, patient demographics, or data quality.
(2) Binary classification model: Currently, the ECCADx system is limited to a binary classification task, distinguishing only between AEH/EC and non-cancerous conditions. While this provides crucial initial differentiation, it inherently oversimplifies the complex diagnostic landscape encountered in real-world clinical practice. Accurate diagnosis often requires a more granular distinction among various pathological subtypes. For instance, benign conditions encompass diverse types like simple hyperplasia, complex hyperplasia without atypia, polyps, or submucosal myomas, each with distinct clinical implications. Furthermore, EC itself comprises various histological types and grades, which are critical for determining prognosis and guiding precise treatment strategies.
(3) Retrospective study design: The application and assessment of ECCADx should be conducted in multicenter prospective studies to ensure broader validation in the future.

To address the complexity of real-world hysteroscopic diagnosis, we aim to extend ECCADx to support multi-class classification, enabling the accurate differentiation of a wider spectrum of endometrial pathologies beyond binary classification. Secondly, to further bolster the system’s robustness and clinical utility, we plan to conduct prospective, randomized multicenter studies. This will allow for a more rigorous evaluation of ECCADx’s performance and generalizability across diverse patient populations and clinical environments, paving the way for its eventual clinical implementation.

## Resource availability

### Lead contact

Further information and requests for resources should be directed to and will be fulfilled by the lead contact, Xin Zhu (zhu.xin@tmd.ac.jp).

### Materials availability

No unique reagents or physical materials were generated in this study.

### Data and code availability

• Data: The datasets analyzed in this study are currently unavailable due to ethical approval constraints and data security considerations. However, accession numbers for publicly available datasets (CP-CHILD dataset: https://figshare.com/articles/dataset/CP-CHILD_zip/12554042, PolypGen dataset: https://www.synapse.org/#!Synapse:syn26376615/wiki/613312, IPCL dataset: https://www.synapse.org/#!Synapse:syn21636566/wiki/601346, Hyper Kvasir dataset: https://github.com/simula/hyper-kvasir) are listed in the key resources table.
• Code: The custom code developed for the ECCADx system used in this study is currently unavailable due to associated engineering complexity and proprietary considerations.
• Other items: Any additional information required to reanalyze the data reported in this paper is available from the lead contact upon request.

## Acknowledgments

I would like to express my sincere gratitude to the following doctors for their invaluable contributions to this study: Dr. Wenqing Ma, Dr. Man Wang, Dr. Wei Yan, Dr. Jia Wei, Dr. Wei Shen, Dr. Yueyue Xi, Dr. Su Zhou, Dr. Yan Zhang, Dr. Suzhen Yuan, Dr. Jingyi Wen, Dr. Tian Wang, and Dr. Ming Yuan. Their expertise and dedication in performing the dataset evaluations were instrumental in validating the comparative experiments for my model. I deeply appreciate their commitment to this research, which has significantly enriched the study’s findings. Additionally, I would like to acknowledge the support received that is not covered by the author contribution or funding sections, including administrative and technical assistance, as well as donations in kind, such as materials used in the experiments.

Prof. Yan Wang made significant contributions to data curation. Due to her passing before the manuscript was finalized, she is not listed as an author, but her contributions are sincerely appreciated.

This study was partially supported by the National Key Research and Development Program of China (grant number 2022YFC2704100) and Knowledge Innovation Program of Wuhan Basic Research (No. 2023020201010041).

## Author contributions

Wenwen Wang, X.Z., and S.W. designed the study and the study concept. Wenwen Wang, Y.C., Z.G., A.Z., and X.Z. designed the methodology. Y.C., Z.G., A.Z., and X.Z. designed software. W.M. and X.D. performed validation. Wenwen Wang, Y.C., and S.W. conducted formal analysis. Wenwen Wang, Y.C., Z.G., Wuliang Wang, X.Z., and S.W. performed investigation. Wenwen Wang, W.M., Wuliang Wang, and S.W. performed data curation. Wenwen Wang, Y.C., and Z.G. prepared the original draft with subsequent contributions from X.Z. and X.D. All authors have reviewed and approved this article, and all necessary steps have been taken to maintain the integrity of the work. The authors declare no conflict of interest in relation to the submission of this manuscript.

## Declaration of interests

The authors declare no competing interests.

## STAR★Methods

### Key resources table

**Table:** 

REAGENT or RESOURCE	SOURCE	IDENTIFIER
**Deposited data**		

CP-CHILD Dataset	Hunan Children’s Hospital, China	url: https://figshare.com/articles/dataset/CP-CHILD_zip/12554042
PolypGen Dataset	Multi-center collaboration (Poland, UK, Italy, Norway, Egypt)	url: https://www.synapse.org/#!Synapse:syn26376615/wiki/613312
IPCL Dataset	Magnification endoscopy studies from Taiwan, China	url: https://www.synapse.org/#!Synapse:syn21636566/wiki/601346
Hyper Kvasir Dataset	Simula Research Laboratory, Norway	url: https://github.com/simula/hyper-kvasir

**Software and algorithms**		

Python	Python Software Foundation	Version: 3.9.19; URL: https://www.python.org/
matplotlib	Matplotlib Development Team	Version: 3.9.2; URL: https://matplotlib.org/
numpy	NumPy Developers/NumFOCUS	Version: 1.26.4; URL: https://numpy.org/
opencv-python	OpenCV Development Team	Version: 4.11.0.86; URL: https://opencv.org/
pandas	pandas Development Team/PyData Development Team	Version: 2.2.3; URL: https://pandas.pydata.org/
Pillow	Pillow Developers	Version: 10.4.0; URL: https://python-pillow.org/
scikit-learn	scikit-learn Developers/INRIA	Version: 1.5.2; URL: https://scikit-learn.org/
scipy	SciPy Developers/NumFOCUS	Version: 1.13.1; URL: https://scipy.org/
statsmodels	Statsmodels Developers	Version: 0.14.3; URL: https://www.statsmodels.org/
timm	Ross Wightman (PyTorch Image Models)	Version: 1.0.9; URL: https://github.com/huggingface/pytorch-image-models
torch	PyTorch Team/Facebook AI Research (FAIR)	Version: 2.4.1; URL: https://pytorch.org/
ResNet-50	Kaiming He et al.^17^	URL: https://github.com/pytorch/vision/blob/main/torchvision/models/resnet.py
SimCLR	Chen et al.^12^	URL: https://github.com/sthalles/SimCLR

### Experimental model and study participant details

This multicohort retrospective study was conducted across three tertiary hospitals and adhered to the Declaration of Helsinki. The study protocol received approval from the Medical Ethics Committee of Tongji Hospital Affiliated in Tongji Medical College, Huazhong University of Science and Technology (Approval No. TJ-IRB20190604; Date: June 10th, 2019), the Medical Ethics Committee of Maternal and Child Hospital of Hubei Province (Approval No. [2022] IEC (007); Date: Feb. 10th, 2022), and was recorded at the Institutional Review Board of the Second Affiliated Hospital of Zhengzhou University (Approval No. 2022336; Date: May 31st, 2022). Patient consent was waived because the study adhered to privacy policies, and all training and analyses were conducted using anonymized data.

This study enrolled a total of 1394 patients, contributing 55,874 hysteroscopy images in PNG format. All images were confirmed by two expert professionals, W.W.W. and W.M. The AEH/EC (atypical endometrial hyperplasia and endometrial cancer) categories included both atypical endometrial hyperplasia and endometrial cancer cases. The dataset was stratified into a training set for model development (comprising 1204 patients and 49,646 images) and two independent test sets. These test sets included an internal cohort from MCH (85 patients, 3419 images) and an external cohort from TJH/ZZSH (105 patients, 2809 images). The control group, comprising benign lesions, encompassed cases with endometrial polyps, submucosal uterine leiomyoma, endometrial hyperplasia without atypia, and normal uterine cavities. Crucially, there was no overlap of cases between the training and test datasets. Table 1 provides a detailed summary of the clinical demographic characteristics and image composition of patients across the training and the two independent test datasets. Further detailed information on various non-cancerous disorders within the control group is provided in Table S7.

The training dataset was collected from January 2008 to December 2017 at the Maternal and Child Hospital of Hubei Province (MCH) using Olympus OTV-S190 (Japan) and Karl Storz 26105FA or 26120BA (Germany) devices. The internal test dataset consisted of images acquired between January 2018 and June 2019 at MCH using the same devices. The external test dataset, referred to as TJH/ZZSH, comprised data collected from January 2019 to December 2019 at Tongji Hospital of Huazhong University of Science and Technology (TJH) and the Second Affiliated Hospital of Zhengzhou University (ZZSH). The AEH/EC categories included cases of atypical endometrial hyperplasia and endometrial cancer. The external test dataset was predominantly obtained using the Olympus OTV-S190 (Japan). There was no overlap of cases between the training and test datasets.

The full dataset was divided into a training dataset and two independent test datasets (MCH internal test dataset and TJH/ZZSH external test dataset), ensuring no overlap of cases between the training and test sets. The detailed numbers of cases and images for each dataset are listed in Table 1: MCH Training Dataset: Collected from January 2008 to December 2017 at the Maternal and Child Hospital of Hubei Province (MCH). This dataset included 106 AEH/EC cases (3204 images) and 1098 control cases (46,442 images). Images were acquired using both Olympus OTV-S190 (Japan) and Karl Storz 26105FA or 26120BA (Germany) devices. MCH Internal Test Dataset: Consisted of 23 AEH/EC cases (698 images) and 62 control cases (2721 images). These images were acquired between January 2018 and June 2019 at MCH using the same devices as the training set. TJH/ZZSH External Test Dataset: Included 16 AEH/EC cases (760 images) and 89 control cases (2049 images). Data for this dataset was collected from January 2019 to December 2019 at Tongji Hospital of Huazhong University of Science and Technology (TJH) and the Second Affiliated Hospital of Zhengzhou University (ZZSH). This external dataset was predominantly obtained using the Olympus OTV-S190 (Japan).

For the human expert evaluation, twelve gynecological endoscopists were enlisted. This group included four junior endoscopists (less than one year of experience), four intermediate endoscopists (1–5 years of experience), and four senior endoscopists (over 10 years of experience). The MCH test dataset was evaluated by endoscopists from TJH, while the TJH/ZZSH dataset was assessed by endoscopists from MCH. Each endoscopist assessed the images for each patient, classifying them based on their clinical expertise into categories such as “Definitely benign,” “Most likely benign,” “Possibly benign,” “Possibly malignant,” “Most likely malignant,” or “Definitely malignant”. All extracted images from the test datasets were utilized to assess the performance of both ECCADx and the endoscopists.

### Method details

#### ECCADx system design overview

Figure 1 provides an overview of the complete workflow for the ECCADx system, spanning data acquisition, model pre-training, classification model development, and final validation. The system initiates with CL pre-training using a large-scale colonoscopy image dataset to extract high-quality features. Subsequently, the pre-trained model is fine-tuned on a hysteroscopy image dataset for classification model development, leveraging supervised learning to achieve precise classification of endometrial lesions. Finally, the system’s performance undergoes rigorous evaluation on independent internal and external test sets, with its performance further compared against multi-level expert assessments.

#### Data preprocessing and augmentation

All hysteroscopy images were uniformly resized to 224x224 pixels. To ensure consistency and enhance model robustness across diverse endoscopic imaging conditions, a comprehensive set of data augmentation techniques was applied. These augmentations included conventional transformations such as random cropping (followed by resizing and horizontal flipping), random color distortions (adjusting brightness, contrast, and saturation), and random Gaussian blur.

Furthermore, to adapt the model more effectively to the specific nuances and challenges of hysteroscopy images and bridge the domain gap from colonoscopy pre-training, several domain-specific augmentations were incorporated. These included simulating realistic variations in lighting and brightness, applying random color cast adjustments to account for different equipment and physiological fluid influences, introducing simulated instrument occlusion, random black patches mimicking surgical tools. Additionally, mild geometric distortions were applied to account for potential lens aberrations or slight scope movements. This strategic combination of conventional and domain-adaptive augmentations aimed to train a highly generalizable and robust model for endometrial lesion classification.

#### ResNet-50 model architecture and pre-training

In this study, a convolutional neural network (CNN) based on the ResNet-50 architecture was designed as the backbone for the analysis of hysteroscopic images.^17^ ResNet-50, a 50-layer deep CNN, is well-suited for this task due to its residual connections, which effectively mitigate the vanishing gradient problem and facilitate the training of deeper networks. The model was initially pre-trained on the ImageNet database, comprising over 100 million images, providing a robust foundation for transfer learning in medical image analysis.^23^

#### Contrastive learning pre-training

Following ImageNet pre-training, ECCADx’s feature extraction was further refined in a second, self-supervised CL phase using the SimCLR framework.^12^ This pre-training was performed on four publicly available external colonoscopy datasets (CP-CHILD,^24^ PolypGen,^25^^,^^26^^,^^27^ IPCL,^28^ and Hyper Kvasir^29^). While distinct in anatomical context, these datasets share fundamental imaging characteristics with hysteroscopy, such as luminal views, tissue textures, and lesion patterns, allowing the model to learn robust low-level and mid-level visual features transferable across endoscopic modalities. This cross-domain pre-training strategy was designed to provide a strong initialization, and its effectiveness was further enhanced by the aforementioned domain-specific augmentations during training. Crucially, SimCLR directly addresses the scarcity of labeled hysteroscopic images. It learns highly discriminative feature representations by maximizing agreement between different augmented views of the same image, while pushing representations of distinct images apart in the latent space using the NT-Xent loss. Diverse augmentations are vital, fostering invariance to transformations and ensuring robust, transferable features for the downstream AEH/EC classification task.

The model architecture comprised a ResNet-50 backbone followed by a two-layer Multi-Layer Perceptron (MLP) projection head. The projection head transformed the 2048-dimensional output features from the ResNet-50 backbone into a 2048-dimensional hidden layer, then projected them to a 128-dimensional latent space for contrastive loss calculation. Training was conducted on a system equipped with four NVIDIA A800 80GB GPUs. The AdamW optimizer was employed with an initial learning rate of 3e-4 and a weight decay of 1e-5. A cosine annealing learning rate schedule was utilized. Training was performed with a global batch size of 2048 (distributed across the four GPUs) for 300 epochs. The normalized temperature-scaled cross-entropy (NT-Xent) loss function was used, with the temperature parameter (τ) set to 0.07. To prevent information leakage during distributed training, Global Batch Normalization was implemented, aggregating batch statistics across all devices.

#### Fine-tuning parameters and strategies

After ImageNet and self-supervised contrastive pre-training, ECCADx fine-tunes its pre-trained ResNet-50 backbone for task-specific AEH/EC classification. In this final stage, the backbone acts as the primary feature extractor, with layers strategically managed to optimize performance and preserve fundamental visual understanding.

During the fine-tuning process, only the initial convolutional layer (conv1) and its corresponding batch normalization layer (bn1) of the pre-trained ResNet-50 network were frozen. This strategic freezing allowed the remaining parameters to be updated, enabling more extensive adaptation of the learned features to the specific characteristics of hysteroscopy images while retaining robust low-level feature extraction capabilities.

The same GPU setup was utilized for fine-tuning. The Stochastic Gradient Descent (SGD) optimizer with a momentum of 0.9 was employed. An initial learning rate of 1e-4 was set, accompanied by a cosine annealing learning rate schedule. Training was conducted for 50 epochs with a global batch size of 4096.

To address the inherent class imbalance within the dataset, specifically the fewer cases of malignancies (AEH/EC), the Focal Loss function was adopted.^30^ Focal Loss multiplies the standard cross-entropy function with a modulating factor to increase the sensitivity toward misclassified AEH/EC observations. This was complemented by an oversampling strategy to further compensate for data imbalance in the training dataset.^31^ The optimal classification threshold was determined by maximizing the F1 score on a dedicated validation set.

### Quantification and statistical analysis

Model performance on both the internal and external test datasets was evaluated using a comprehensive set of metrics. These included Accuracy, Sensitivity, Specificity, Precision, Recall, F1 score, and the Area Under the Receiver Operating Characteristic Curve (AUC). For all reported metrics, 95% confidence intervals (CIs) were calculated.

Statistical analyses were performed to compare diagnostic performances. For paired comparisons of diagnostic models (e.g., comparing ECCADx with individual human experts), McNemar’s chi-square test was employed. For unpaired comparisons of diagnostic models (e.g., comparing groups of experts or different versions of the model if applicable in results), Fisher’s exact test was utilized. The AUCs of ECCADx and each human expert were specifically compared using Delong’s test to assess statistically significant differences in diagnostic accuracy. Statistical significance for all analyses was set at a two-sided *p*-value of less than 0.05.
