# Supplementary material for: A deep learning-based computer-aided diagnosis system for detecting atypical endometrial hyperplasia and endometrial cancer through hysteroscopy
Source: iScience. 2025 Jul 3;28(8):113045. doi: 10.1016/j.isci.2025.113045 (PMC12283552; doi:10.1016/j.isci.2025.113045)
Supplement: Document S1. Figures S1 and S2 and Tables S1–S7 [file mmc1.pdf]

iScience, Volume *28*

Supplemental information

A deep learning-based computer-aided diagnosis system for detecting atypical endometrial hyperplasia and endometrial cancer through hysteroscopy

Wenwen Wang, Yuyang Cai, Zhe Guo, Aihua Zhao, Wenqing Ma, Wuliang Wang, Shixuan Wang, Xin Zhu, Xin Du, and Wenfeng Shen

Supplementary Tables

Table S1: Performance Evaluation of Junior Level Endoscopists in the MCH test Dataset, Related to Table 2.

	Gynecological endoscopist				ECCADx	ECCADx
	Junior-Exp1	Junior-Exp2	Junior-Exp3	Junior-Exp4	(without CL)	(with CL)
AUC (95% CI)	0.877 (0.796-0.943)	0.884 (0.808-0.950)	0.778 (0.674-0.876)	0.948 (0.896-0.984)	0.969 (0.928-0.999)	0.979 (0.942-1.000)
P value*	0.839	0.0086	0.0012	0.241	0.175	N/A
Accuracy (95% CI)	81.2% (72.9-89.5%)	87.1% (79.9-94.2%)	81.2% (72.9-89.5%)	84.7% (77.0-92.4%)	91.8% (85.9-97.6%)	94.1% (89.1-99.1%)
Sensitivity (95% CI)	60.9% (40.9-81.0%)	82.6% (66.6-95.7%)	56.5% (35.0-76.0%)	95.7% (85.2-100%)	96.8% (91.7-100%)	95.2% (89.5-100%)
Specificity (95% CI)	88.7% (80.0-95.5%)	88.7% (80.7-95.5%)	90.3% (82.5-96.8%)	80.7% (70.7-90.0%)	78.3% (60.9-92.9%)	91.3% (78.2-100%)
PPV (95% CI)	66.7% (45.8-85.7%)	73.1% (56.0-88.9%)	68.4% (46.7-90.0%)	64.7% (48.6-80.7%)	92.3% (84.9-98.4%)	96.7% (91.8-100%)
NPV (95% CI)	85.9% (76.2-93.9%)	93.2% (86.6-98.4%)	84.9% (76.4-93.8%)	98.0% (92.9-100%)	90.0% (75.0-100%)	87.5% (72.7-100%)
F1 (95% CI)	0.636 (0.437-0.783)	0.775 (0.622-0.884)	0.619 (0.414-0.773)	0.772 (0.631-0.879)	0.945 (0.902-0.979)	0.959 (0.920-0.992)
Kappa (95% CI)	0.510 (0.304-0.704)	0.685 (0.510-0.844)	0.495 (0.268-0.694)	0.663 (0.507-0.826)	0.782 (0.601-0.916)	0.853 (0.712-0.969)
Brier (95% CI)	0.128 (0.081-0.176)	0.160 (0.139-0.183)	0.174 (0.149-0.197)	0.113 (0.078-0.151)	0.060 (0.033-0.093)	0.040 (0.014-0.075)

* DeLong's test (Exp and ECCADx without CL vs ECCADx with CL)

Table S2: Performance Evaluation of Medium Level Endoscopists in the MCH test Dataset, Related to Table 2.

	Gynecological endoscopist				ECCADx	ECCADx
	Medium-Exp1	Medium-Exp2	Medium-Exp3	Medium-Exp4	(without CL)	(with CL)
AUC (95% CI)	0.964 (0.927-0.991)	0.957 (0.915-0.987)	0.917 (0.849-0.967)	0.897 (0.819-0.957)	0.969 (0.928-0.999)	0.979 (0.942-1.000)
P value*	0.509	0.348	0.031	0.058	0.175	N/A
Accuracy (95% CI)	92.9% (87.5-98.4%)	88.2% (81.4-95.1%)	84.7% (77.0-92.4%)	85.9% (78.5-93.3%)	91.8% (85.9-97.6%)	94.1% (89.1-99.1%)
Sensitivity (95% CI)	95.7% (85.2-100%)	65.2% (45.5-84.6%)	65.2% (44.4-84.6%)	65.2% (45.0-85.0%)	96.8% (91.7-100%)	95.2% (89.5-100%)
Specificity (95% CI)	91.9% (84.2-98.3%)	96.8% (91.7-100%)	91.9% (84.8-98.4%)	93.5% (86.9-98.5%)	78.3% (60.9-92.9%)	91.3% (78.2-100%)
PPV (95% CI)	81.5% (65.4-95.2%)	88.2% (71.4-100%)	75.0% (53.8-93.3%)	79.0% (58.3-95.2%)	92.3% (84.9-98.4%)	96.7% (91.8-100%)
NPV (95% CI)	98.3% (94.2-100%)	88.2% (80.0-95.5%)	87.7% (79.7-95.2%)	87.9% (80.0-95.5%)	90.0% (75.0-100%)	87.5% (72.7-100%)
F1 (95% CI)	0.880 (0.772-0.964)	0.750 (0.578-0.881)	0.698 (0.540-0.842)	0.714 (0.538-0.849)	0.945 (0.902-0.979)	0.959 (0.920-0.992)
Kappa (95% CI)	0.831 (0.681-0.948)	0.675 (0.473-0.845)	0.596 (0.385-0.781)	0.622 (0.412-0.804)	0.782 (0.601-0.916)	0.853 (0.712-0.969)
Brier (95% CI)	0.104 (0.083-0.128)	0.091 (0.066-0.115)	0.107 (0.076-0.141)	0.111 (0.078-0.148)	0.060 (0.033-0.093)	0.040 (0.014-0.075)

Table S3: Performance Evaluation of Senior Level Endoscopists in the MCH test Dataset, Related to Table 2.

	Gynecological endoscopist				ECCADx	ECCADx
	Senior-Exp1	Senior-Exp2	Senior-Exp3	Senior-Exp4	(without CL)	(with CL)
AUC (95% CI)	0.983 (0.961-0.996)	0.962 (0.920-0.993)	0.952 (0.904-0.983)	0.913 (0.821-0.972)	0.969 (0.928-0.999)	0.979 (0.942-1.000)
P value*	0.017	0.485	0.279	0.096	0.175	N/A
Accuracy (95% CI)	94.1% (89.1-99.1%)	92.9% (87.5-98.4%)	89.4% (82.9-96.0%)	87.1% (79.9-94.2%)	91.8% (85.9-97.6%)	94.1% (89.1-99.1%)
Sensitivity (95% CI)	100% (100-100%)	100% (100-100%)	78.3% (60.0-94.1%)	69.6% (50.0-87.5%)	96.8% (91.7-100%)	95.2% (89.5-100%)
Specificity (95% CI)	91.9% (84.9-98.3%)	90.3% (82.1-96.8%)	93.5% (87.1-98.5%)	93.5% (87.0-98.5%)	78.3% (60.9-92.9%)	91.3% (78.2-100%)
PPV (95% CI)	82.1% (65.5-95.8%)	79.3% (64.0-92.9%)	81.8% (65.0-96.0%)	80.0% (60.0-100%)	92.3% (84.9-98.4%)	96.7% (91.8-100%)
NPV (95% CI)	100% (100-100%)	100% (100-100%)	92.1% (85.2-98.4%)	89.2% (81.0-95.7%)	90.0% (75.0-100%)	87.5% (72.7-100%)
F1 (95% CI)	0.902 (0.800-0.980)	0.885 (0.783-0.962)	0.800 (0.652-0.917)	0.744 (0.571-0.865)	0.945 (0.902-0.979)	0.959 (0.920-0.992)
Kappa (95% CI)	0.861 (0.740-0.969)	0.835 (0.693-0.946)	0.728 (0.542-0.884)	0.658 (0.452-0.828)	0.782 (0.601-0.916)	0.853 (0.712-0.969)
Brier (95% CI)	0.065 (0.046-0.085)	0.070 (0.027-0.120)	0.085 (0.060-0.111)	0.103 (0.071-0.143)	0.060 (0.033-0.093)	0.040 (0.014-0.075)

Table S4: Performance Evaluation of Junior Level Endoscopists in the TJH/ZZSH test datasets, Related to Table 3.

	Gynecological endoscopist				ECCADx	ECCADx
	Junior-Exp1	Junior-Exp2	Junior-Exp3	Junior-Exp4	(without CL)	(with CL)
AUC (95% CI)	0.722 (0.570-0.843)	0.780 (0.665-0.875)	0.704 (0.600-0.808)	0.886 (0.802-0.945)	0.891 (0.810-0.964)	0.975 (0.942-0.998)
P value*	0.0024	0.0013	0.0017	0.021	0.028	N/A
Accuracy (95% CI)	78.1% (70.2-86.0%)	73.3% (64.9-81.8%)	81.0% (73.4-88.5%)	74.3% (65.9-82.7%)	89.5% (83.7-95.4%)	93.3% (88.6-98.1%)
Sensitivity (95% CI)	50.0% (25.0-75.0%)	81.2% (60.0-100%)	37.5% (14.3-62.5%)	93.8% (80.0-100%)	94.4% (89.4-98.8%)	92.1% (86.4-96.8%)
Specificity (95% CI)	83.2% (74.7-90.4%)	71.9% (61.9-81.1%)	88.8% (81.8-94.7%)	70.8% (61.4-80.0%)	62.5% (40.0-86.7%)	100% (100.0-100%)
PPV (95% CI)	34.8% (16.7-57.1%)	34.2% (18.9-50.0%)	37.5% (14.3-63.2%)	36.6% (21.2-50.0%)	93.3% (87.9-97.8%)	100% (100.0-100%)
NPV (95% CI)	90.2% (83.7-96.2%)	95.5% (89.8-100%)	88.8% (81.7-94.6%)	98.4% (95.0-100%)	66.7% (40.0-88.2%)	69.6% (50.0-87.5%)
F1 (95% CI)	0.410 (0.188-0.596)	0.481 (0.292-0.638)	0.375 (0.154-0.585)	0.526 (0.363-0.667)	0.939 (0.898-0.973)	0.959 (0.925-0.988)
Kappa (95% CI)	0.281 (0.058-0.480)	0.340 (0.154-0.509)	0.263 (0.022-0.486)	0.393 (0.223-0.557)	0.584 (0.330-0.780)	0.781 (0.607-0.926)
Brier (95% CI)	0.160 (0.112-0.213)	0.212 (0.188-0.238)	0.178 (0.157-0.201)	0.185 (0.149-0.226)	0.103 (0.068-0.141)	0.072 (0.044-0.109)

Table S5: Performance Evaluation of Medium Level Endoscopists in the TJH/ZZSH test datasets, Related to Table 3.

	Gynecological endoscopist				ECCADx	ECCADx
	Medium-Exp1	Medium-Exp2	Medium-Exp3	Medium-Exp4	(without CL)	(with CL)
AUC (95% CI)	0.785 (0.653-0.891)	0.772 (0.644-0.895)	0.578 (0.441-0.707)	0.833 (0.699-0.927)	0.891 (0.810-0.964)	0.975 (0.942-0.998)
P value*	0.0017	0.0024	0.024	0.000	0.028	N/A
Accuracy (95% CI)	73.3% (64.9-81.8%)	82.9% (75.6-90.1%)	69.5% (60.7-78.3%)	83.8% (76.8-90.9%)	89.5% (83.7-95.4%)	93.3% (88.6-98.1%)
Sensitivity (95% CI)	75.0% (52.9-94.4%)	62.5% (38.5-87.0%)	25.0% (5.3-46.7%)	68.8% (44.4-91.7%)	94.4% (89.4-98.8%)	92.1% (86.4-96.8%)
Specificity (95% CI)	73.0% (64.1-81.8%)	86.5% (78.9-93.2%)	77.5% (68.5-85.9%)	86.5% (79.2-93.6%)	62.5% (40.0-86.7%)	100% (100.0-100%)
PPV (95% CI)	33.3% (18.7-51.4%)	45.5% (25.0-66.7%)	16.7% (3.7-33.3%)	47.8% (25.9-68.8%)	93.3% (87.9-97.8%)	100% (100.0-100%)
NPV (95% CI)	94.2% (88.6-98.6%)	92.8% (86.9-97.7%)	85.2% (76.5-92.5%)	93.9% (88.5-98.7%)	66.7% (40.0-88.2%)	69.6% (50.0-87.5%)
F1 (95% CI)	0.462 (0.280-0.615)	0.526 (0.333-0.708)	0.200 (0.046-0.368)	0.564 (0.333-0.724)	0.939 (0.898-0.973)	0.959 (0.925-0.988)
Kappa (95% CI)	0.318 (0.137-0.504)	0.425 (0.191-0.643)	0.021 (0.000-0.229)	0.469 (0.233-0.667)	0.584 (0.330-0.780)	0.781 (0.607-0.926)
Brier (95% CI)	0.181 (0.151-0.211)	0.146 (0.121-0.172)	0.196 (0.162-0.236)	0.139 (0.114-0.168)	0.103 (0.068-0.141)	0.072 (0.044-0.109)

Table S6: Performance Evaluation of Senior Level Endoscopists in the TJH/ZZSH test datasets, Related to Table 3.

	Gynecological endoscopist				ECCADx	ECCADx
	Senior-Exp1	Senior-Exp2	Senior-Exp3	Senior-Exp4	(without CL)	(with CL)
AUC (95% CI)	0.824 (0.728-0.912)	0.839 (0.755-0.908)	0.893 (0.821-0.956)	0.893 (0.830-0.943)	0.891 (0.810-0.964)	0.975 (0.942-0.998)
P value*	0.0014	0.000	0.030	0.012	0.028	N/A
Accuracy (95% CI)	76.2% (68.0-84.3%)	76.2% (68.0-84.3%)	81.9% (74.5-89.3%)	86.7% (80.2-93.2%)	89.5% (83.7-95.4%)	93.3% (88.6-98.1%)
Sensitivity (95% CI)	75.0% (50.0-93.3%)	75.0% (52.6-94.5%)	81.2% (61.1-100.%)	56.2% (33.3-80.0%)	94.4% (89.4-98.8%)	92.1% (86.4-96.8%)
Specificity (95% CI)	76.4% (67.8-84.6%)	76.4% (67.0-85.2%)	82.0% (73.9-89.0%)	92.1% (85.9-97.0%)	62.5% (40.0-86.7%)	100% (100.0-100%)
PPV (95% CI)	36.4% (20.6-53.3%)	36.4% (20.7-54.2%)	44.8% (27.6-64.5%)	56.2% (31.6-81.0%)	93.3% (87.9-97.8%)	100% (100.0-100%)
NPV (95% CI)	94.4% (88.7-98.7%)	94.4% (89.2-98.7%)	96.0% (91.1-100%)	92.1% (86.0-97.6%)	66.7% (40.0-88.2%)	69.6% (50.0-87.5%)
F1 (95% CI)	0.490 (0.304-0.650)	0.490 (0.298-0.644)	0.578 (0.400-0.746)	0.562 (0.312-0.750)	0.939 (0.898-0.973)	0.959 (0.925-0.988)
Kappa (95% CI)	0.358 (0.156-0.537)	0.358 (0.171-0.549)	0.475 (0.265-0.656)	0.484 (0.228-0.692)	0.584 (0.330-0.780)	0.781 (0.607-0.926)
Brier (95% CI)	0.182 (0.153-0.212)	0.187 (0.130-0.254)	0.139 (0.110-0.173)	0.120 (0.091-0.160)	0.103 (0.068-0.141)	0.072 (0.044-0.109)

Table S7 Information of non-cancerous disorders, Related to Table 1.

	Training dataset	MCH test dataset	TJH/ZZSH test dataset
P	260	21	48
NE	499	41	30
UL	194	0	9
EH	153	0	2

P: polyp; NE: normal endometrium; UL: Uterine Leiomyomata; EH: endometrial hyperplasia.

Supplementary Figures

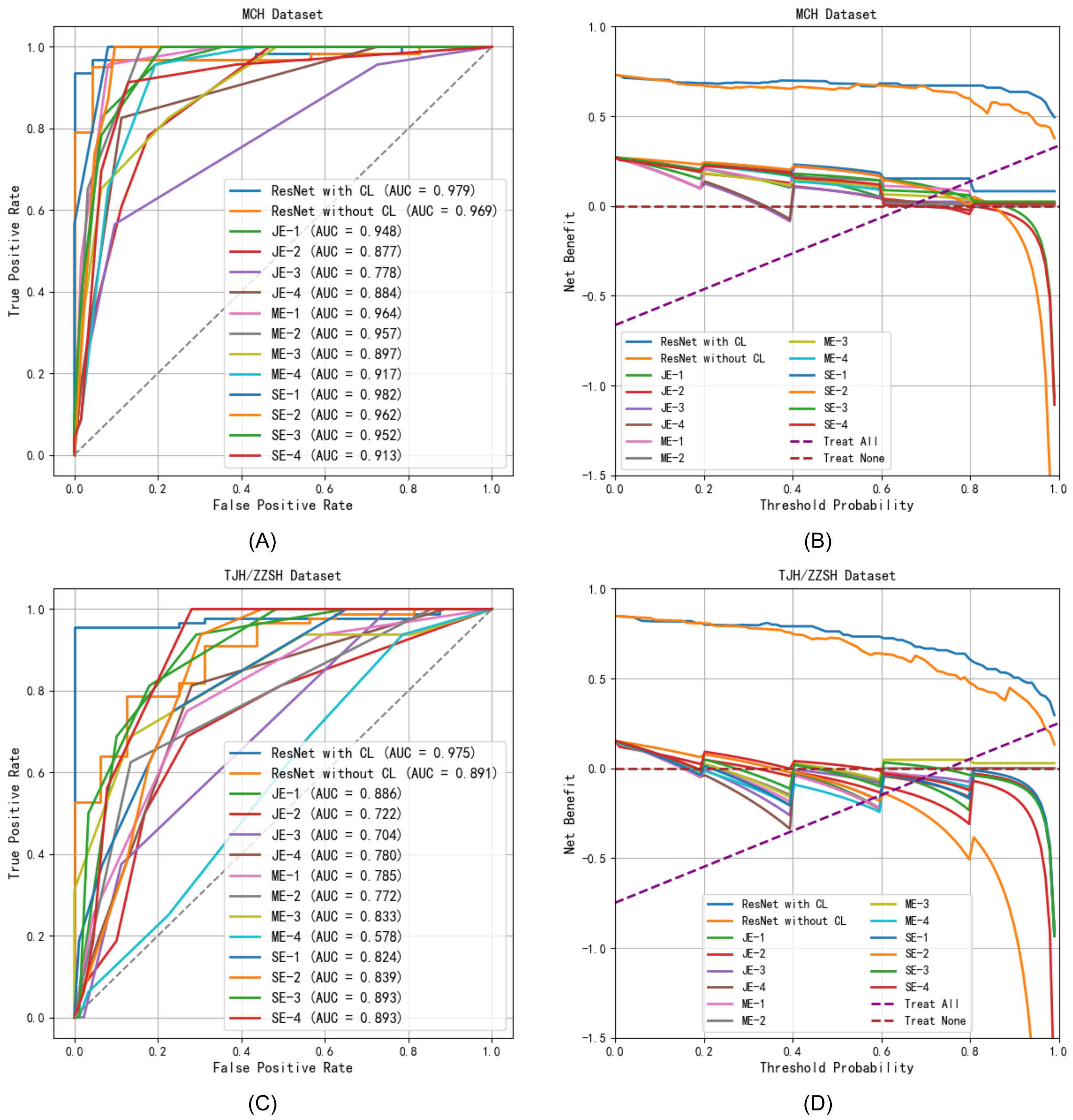

JE: junior endoscopists (<1 year), ME: medium endoscopists (1-5 years), SE: senior endoscopists (>10 years).

Figure S1. Performance Comparison of Different Models on MCH and TJH/ZZSH Datasets, Related to Table 2 and Table 3. (A) ROC Curves for MCH Dataset, illustrating the classification performance of various models on the MCH dataset, with AUC values indicating overall accuracy. (B) Decision Curve Analysis (DCA) for MCH Dataset, displaying the net benefit of different models across various threshold probabilities on the MCH dataset, including "Treat All" and "Treat None" baselines. (C) ROC Curves for TJH/ZZSH Dataset, showing the classification performance of the models on the TJH/ZZSH dataset, with AUC values reflecting their accuracy in this context. (D) Decision Curve Analysis (DCA) for TJH/ZZSH Dataset, presenting the net benefit of the models across varying threshold probabilities on the TJH/ZZSH dataset.

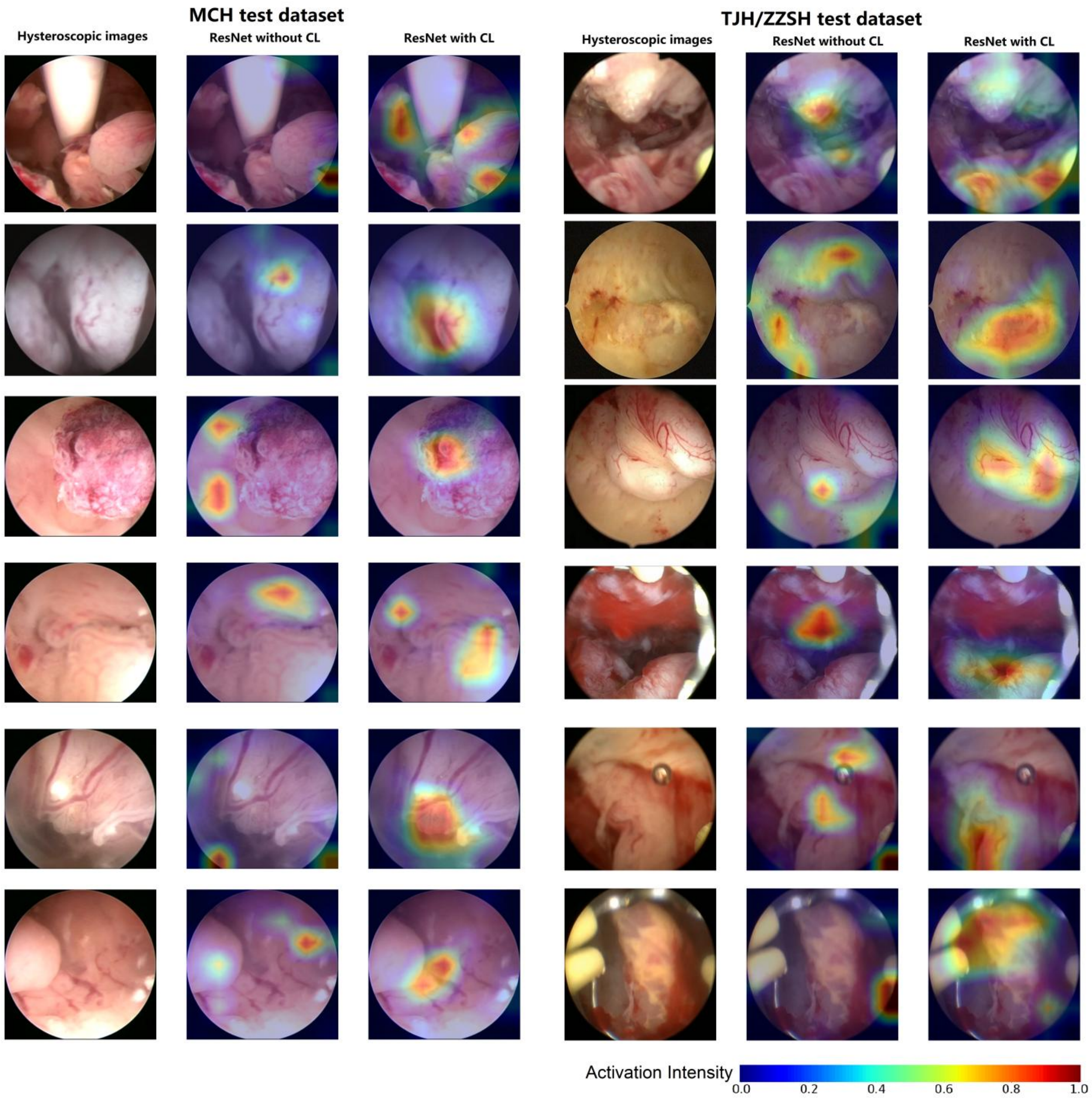

(A)

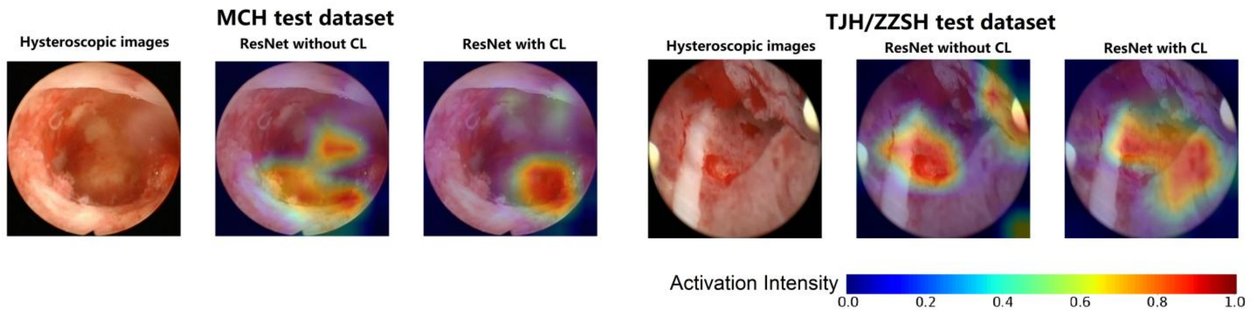

(B)

Figure S2. Comprehensive Grad-CAM Visualizations for ECCADx with and without Contrastive Learning (CL) on Diverse Hysteroscopic Images, Related to Figure 4. (A) This panel presents images depicting cases of AEH/EC. (B) This panel presents images depicting cases of Control.
